# Validation of deep learning enabled web based and smartphone optimized application RadAnalyzer to measure vertebral heart size and vertebral left atrial size in dogs

**DOI:** 10.1371/journal.pone.0337679

**Published:** 2026-05-13

**Authors:** Sonya Gordon, Tomas Reyes, Tabitha Baibos-Reyes, Katharine Tess Sykes, Sukjung Lim, Alice Watson

**Affiliations:** 1 Department of Small Animal Clinical Sciences, Texas A&M University, College Station, Texas, United States of America; 2 RadAnalyzer, Austin, Texas, United States of America; 3 Independent, London, United Kingdom; PRISM CRO, PAKISTAN

## Abstract

**Background:**

Objective radiographic measures of heart size including vertebral heart size (VHS) and vertebral left atrial size (VLAS) are associated with inter and intra-observer variability when measured by humans. Artificial intelligence (AI) tools including RadAnalyzer are available to measure VHS and VLAS.

**Objectives:**

Compare VHS and VLAS measurements made by web based and smartphone optimized deep learning enabled program, RadAnalyzer, to a trained observer.

**Animals:**

High-quality radiographs from 1058 client-owned dogs, across 80 breeds with a variety of heart sizes and thoracic confirmations.

**Methods:**

Retrospective, single center, method comparison study. Pearson’s correlation, Bland-Altman plots and Passing-Bablok regression were used to assess agreement.

**Results:**

RadAnalyzer measurements of VHS and VLAS correlated well with the human observer’s modified measurements (r = 0.917 and r = 0.873 respectively) and had small mean biases (0.002 and 0.007 with limits of agreement of −0.85 to 0.85 and −0.44 to 0.46 vertebrae respectively).

**Conclusions and clinical importance:**

RadAnalyzer had clinically insignificant magnitude differences in measurement of VHS and VLAS when compared to a human observer and can therefore be used to assist veterinarians with measuring VHS and VLAS on good quality right lateral radiographs in dogs of all sizes. Future studies comparing AI derived radiographic measures with echocardiographic measures of cardiac size are required.

## Introduction

Although echocardiography is the gold standard for assessing heart size in dogs [1], echocardiography is not always available. Where echocardiography is not available objective radiographic heart size measurements may be used for staging and monitoring canine cardiac disease [2,3]. Vertebral heart size (VHS) and vertebral left atrial size (VLAS) have been developed to objectively quantify heart size [4,5]. Inter and intra-observer variation has been demonstrated for VHS, with documented differences of up to one vertebra [6,7] which may have significant effects on sequential measurements on the same dog. Similarly, inter and intra observer variability exists for VLAS [8], and may be associated with clinician experience [9]. Despite these limitations, both VHS and VLAS can be helpful in the clinical setting, especially where echocardiography is not feasible.

Artificial intelligence (AI) is being integrated into various aspects of veterinary medicine, including radiographic and cytological interpretation, as well as predictive models of disease [10,11]. Within veterinary cardiology AI has been used to detect cardiomegaly in a binary manner [12,13], and to measure VHS [14,15] and VLAS [16]. A deep learning enabled web based and smartphone optimized application, RadAnalyzer (RadAnalyzer LLC, Austin, USA), was developed to measure VHS and VLAS on lateral thoracic radiographs from dogs. RadAnalyzer is a geometrically informed ensemble of algorithms which was trained on 801 and tested on 199 radiographs. The method used to obtain VHS and VLAS measurements by RadAnalyzer uses an average vertebral size, calculated by taking the mean length of vertebrae four to nine. This modified method positively correlates with the traditional method described by Buchanan and Bucheler [5,17,18]. Measuring VHS and VLAS with AI has the potential to be a fast, easy, and reliable tool for veterinarians but appropriate validation is essential.

This study aimed to compare the performance of a novel algorithm for measuring VHS and VLAS in dogs, RadAnalyzer, to a trained observer for the measurement of VHS and VLAS on a large dataset of canine right lateral radiographs.

## Materials and methods

This was a single site, cross-sectional, retrospective, cohort study. Ethics approval was waived because this was a retrospective study utilizing anonymized radiographs obtained for independent clinical reasons. Right lateral canine thoracic radiographs obtained from a cohort of cases that were collated for another study were measured by the RadAnalyzer application The radiographs were originally reviewed by a board-certified veterinary cardiologist (SG) prior to inclusion and excluded if there was poor image quality, including over- and under-exposure, or if the required VHS anatomical landmarks (carina, apex, heart base, cranial margin of the heart, caudal vena cava, and the fourth and ninth vertebral body) could not be visualized.

### Manual measurements

A single observer (TS) trained by a veterinary cardiologist (SG) measured modified VHS and VLAS (VHS-M and VLAS-M) whereby a mean vertebral length is calculated by measuring from the cranial aspect of T4 to the caudal aspect of T9 and dividing by 5. For VHS the height of the cardiac silhouette was calculated by drawing a line from the center of the ventral aspect of the carina to the ventral most aspect of the cardiac apex and the width of the cardiac silhouette was measured from the ventral aspect of the caudal vena cava to the cranial cardiac waist, perpendicular to the first line. For VLAS a line was drawn from the center of the most ventral aspect of the carina to the most caudal aspect of the left atrium where it intersects with the dorsal border of the caudal vena cava. The number of vertebrae which contribute to the VHS and VLAS were calculated by dividing the sum of the measurements by the mean vertebral length.

### Artificial-Intelligence measurements

**Fig 1:**
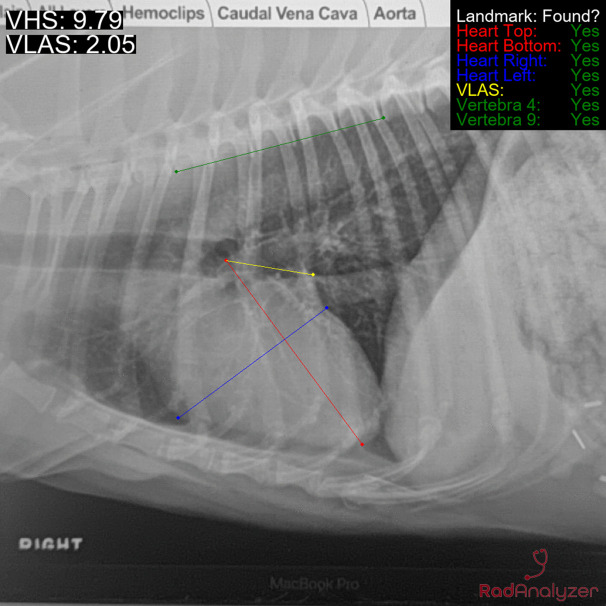
Example of markup by RadAnalyzer algorithm on a right lateral thoracic radiograph. The vertebral heart score is 9.79 and vertebral left atrial score is 2.50. The red line is the long axis of the heart, the blue line is the short axis of the heart, the yellow line is the left atrium, and the green line is from the fourth to ninth vertebra.

RadAnalyzer is a web based and smartphone application. Images of radiographs can be uploaded to the application or photographs of a radiograph are taken within the application which saves and measures the best image and displays the results within 15 seconds. The smartphone application is a unique aspect of this AI radiographic measurement tool. RadAnalyzer assesses images in two stages, stage 1-rough prediction where landmarks are identified and then stage two- a geometrically informed method that fine tunes landmarks identification, images are rejected if prediction is poor. In stage two RadAnalyzer identifies eleven landmarks (carina, cardiac apex, cranial cardiac margin, ventral and dorsal borders of the caudal vena cava as it intersects with the caudal aspect of the cardiac silhouette, the cranial aspect of vertebrae 3, 4 and 5 along and the caudal aspect of vertebrae 8, 9 and 10) for VHS and VLAS calculation (VHS-AI and VLAS-AI, Fig 1).

### Algorithm development and dataset partitioning

RadAnalyzer is a geometrically informed ensemble model, combining learned landmark detection with rule-based geometric constraints derived from established radiographic definitions. Such geometry-aware approaches are well described in the computer vision literature, and ensemble learning is a widely used strategy to improve robustness and accuracy of predictive systems [19,20]. RadAnalyzer was developed using a retrospective dataset of canine right lateral thoracic radiographs that met predefined image quality and anatomical visibility criteria. A total of 1,000 radiographs collected through the RadAnalyzer website were used for algorithm development and evaluation were selected for inclusion by a board-certified cardiologist (SG). A single observer (SL, research intern) trained by a veterinary cardiologist (SG) measured modified VHS and VLAS (VHS-M and VLAS-M). Of these, 801 radiographs were allocated to model training and internal optimization, while 199 radiographs were reserved as an independent test set and were not used during model training or refinement.

### Statistical analysis

Statistical analysis was conducted using R version 4.5.1. Visual inspection of histograms and Shapiro Wilk tests were used to assess the distribution of continuous data. Correlation between methods was assessed using Spearman correlation. Bland-Altman plots [21] were constructed using the *blandr* package and Passing-Bablok regression using the *mcr* package used to assess agreement between methods. Heteroscedasticity and proportional bias of points on the Bland-Altman plots were assessed using Pearson’s correlation and linear regression. The 95th percentile of absolute difference between VHS-M and VHS-AI were calculated and the 95% confidence intervals computed using bootstrapping resampling with replacement using the *boot* package, the number of bootstrap replicates was set to 10,000. The ground truth was defined as the VHS-M and VLAS-M measurements made by a single, trained observer.

## Results

Radiographs from 1058 dogs representing 106 breeds were included in this study with the most common breeds including: Cavalier King Charles Spaniel (28.4%), Doberman Pinscher (7.7%), Labrador Retriever (7.2%) and Boxer (6.3%) remaining breeds contributed less than 5% each (S1 Table). There was an equal distribution of males (51.0%) and females (49.0%), and the majority were sterilized (83.8%). Median age was 9.4 years (IQR, 6.7–11.2 years, range, 0.4–17.3 years), and median body weight was 11.6 kg (IQR, 7.5–30.4 kg, range, 1.6–90.0 kg). The range of VHS-M and VLAS-M measured by a single trained observer were 8.7–15.5 and 1.0–5.0 vertebrae respectively demonstrating that a wide range of cardiac sizes were included in the study.

VHS was measured on all images by the human and RadAnalyzer. It was not possible for the human observer to measure VLAS in 4 cases and RadAnalyzer algorithm to measure VLAS in 73 different cases, meaning a total of 981 cases had both manual and AI measurements of VLAS.

### Comparison of AI and human

**Fig 2:**
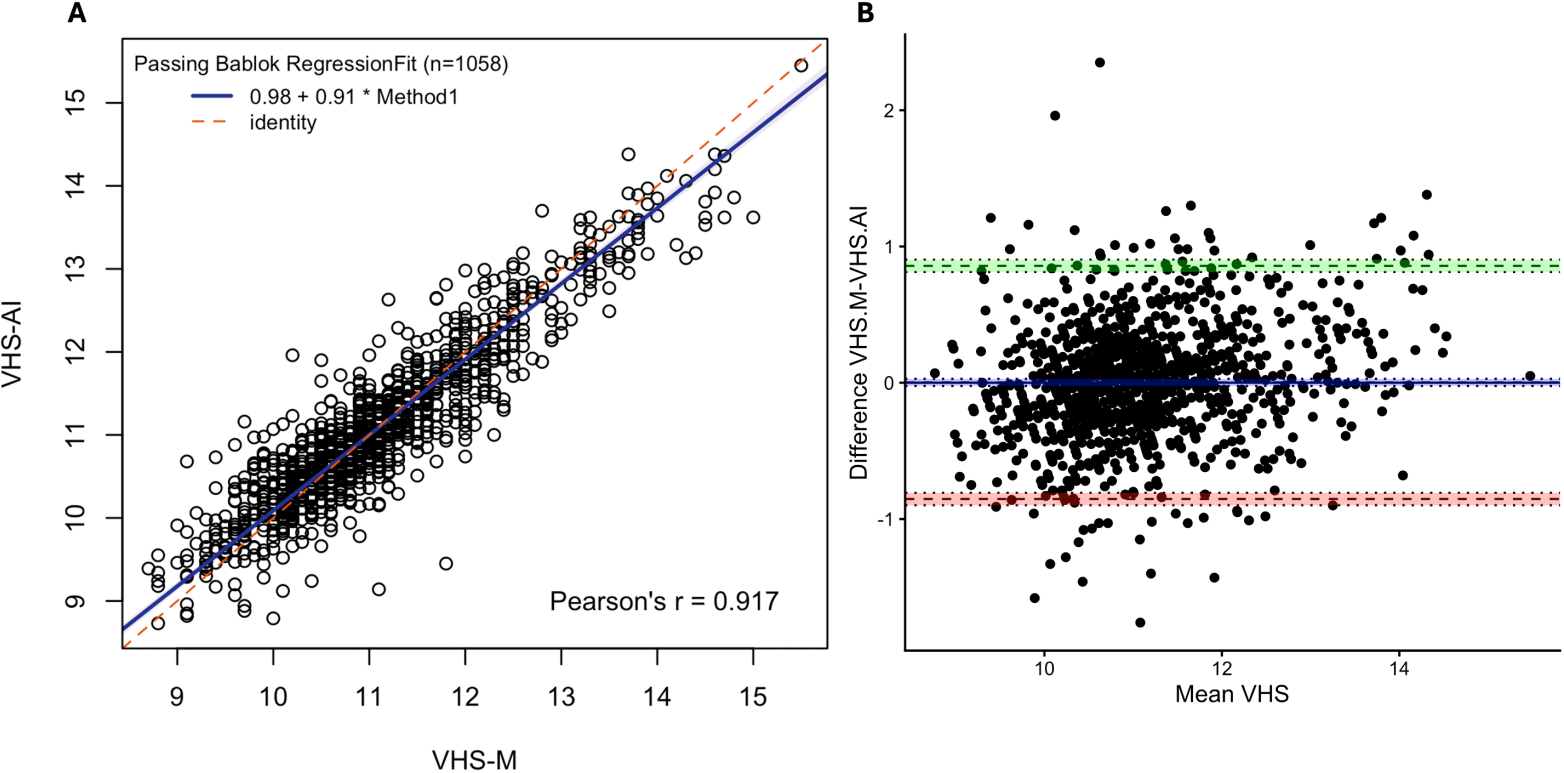
Comparison of VHS measurement by human and RadAnalyzer. Passing–Bablok analysis (A) analysis yielded the equation VHS-AI = 0.91(VHS-M)+0.98, Pearson’s linear correlation coefficient r = 0.917, n = 1058. Blue line: fitted regression line, shading: 95% confidence interval, red dashed line: identity. Bland–Altman analysis (B) demonstrated a mean bias of 0.002 vertebrae (95% CI −0.023 to 0.029) for the VHS-AI method. Dashed lines give limits of agreement (±1.96 SD) and mean bias; shading gives 95% confidence intervals with dotted lines at the limits. VHS indicates vertebral heart size; VHS-AI, VHS measured by RadAnalyzer, VHS-M, VHS measured by a human observer and SD, standard deviation.

**Fig 3:**
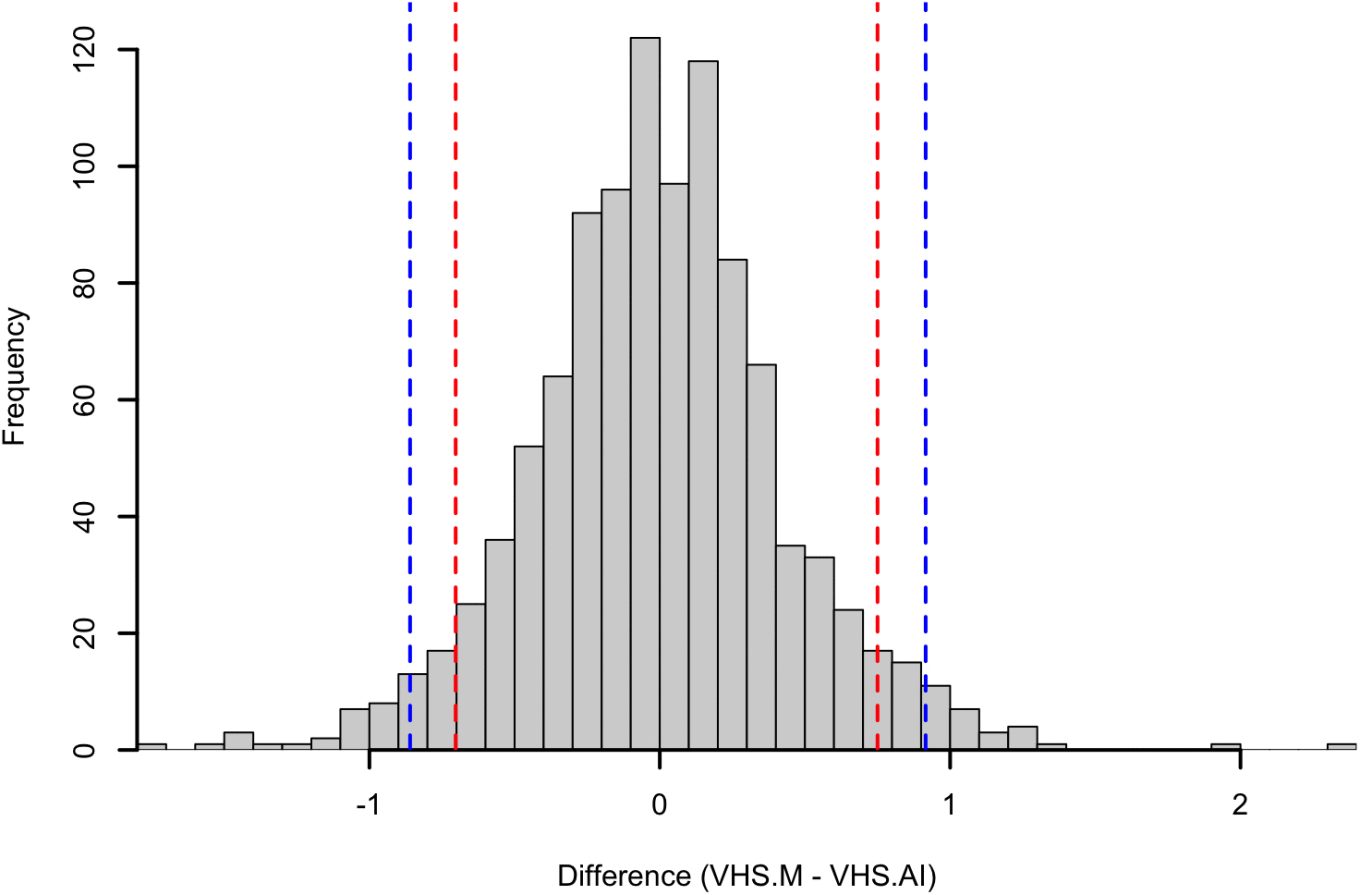
Histogram of the difference between modified vertebral heart size (VHS) determined by human observer (VHS.M) and Radanalyzer (VHS.AI). The red lines indicate the 5th percentile and 95th percentile. The blue dashed lines represent the 2.5th percentile and the 97.5th percentile.

1058 measurements of VHS-M and VHS-AI were available from right lateral radiographic views. For VHS, Pearson’s correlation was 0.917 (95% CI 0.907 to 0.926, p < 0.001). Passing-Bablok analysis (Fig 2A) yielded the equation VHS-AI = 0.91(VHS-M)+0.97, with 95% CI of 0.89–0.93 for the slope and 0.73 to 1.21 for the intercept. Bland–Altman analysis (Fig 2B) showed a bias of 0.002 (SE 0.013; 95% CI −0.024 to 0.029) between VHS-M and VHS-AI with limits of agreement between −0.85 and 0.86 vertebrae although significant heteroscedasticity and proportional biases was observed. The 95^th^ percentile absolute difference between VHS-M and VHS-AI was 0.88 vertebrae (95% CI 0.83–0.97, Fig 3).

**Fig 4:**
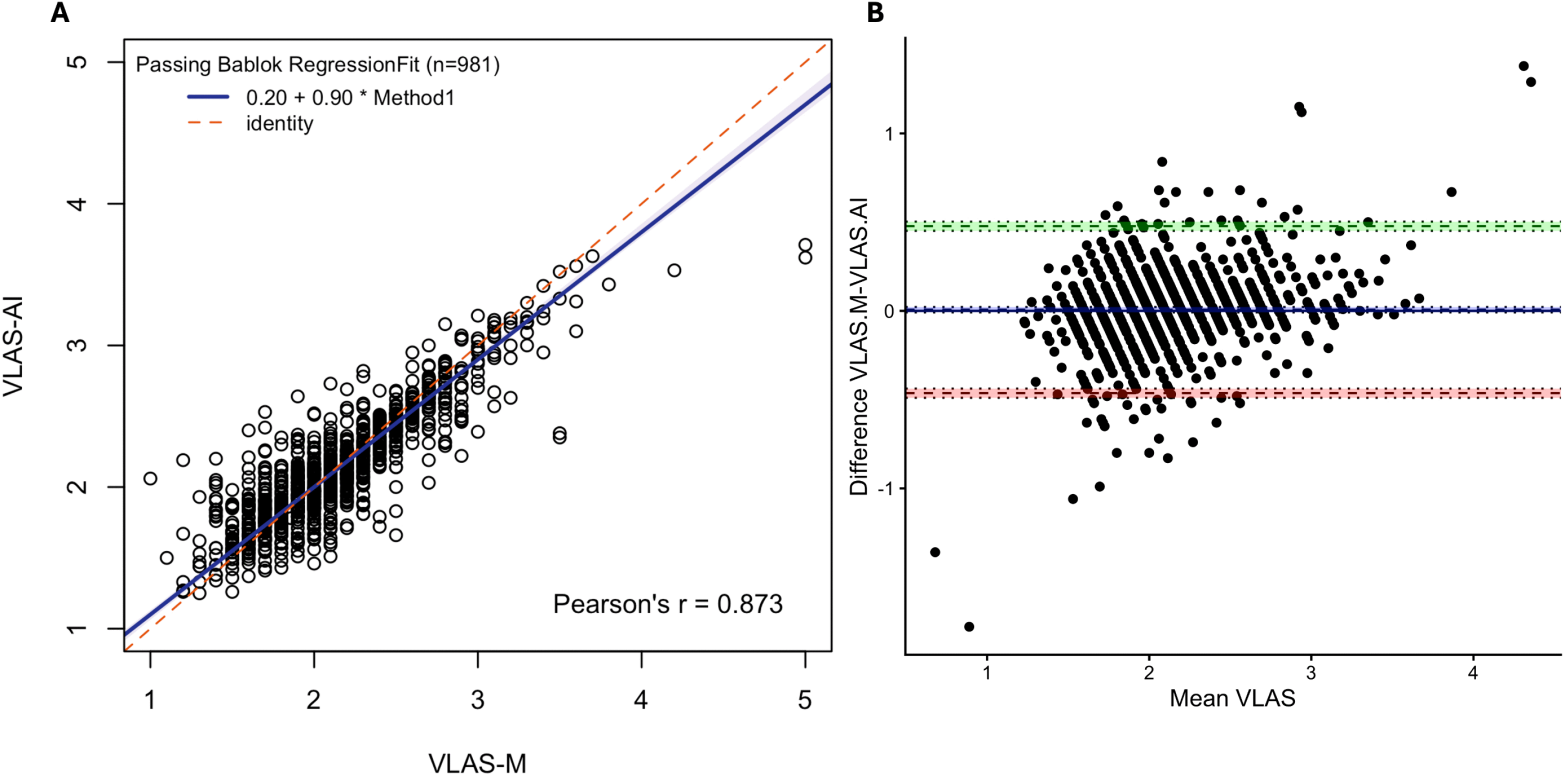
Comparison of VLAS measurement by human and RadAnalyzer. Passing–Bablok analysis. (A) yielded the equation VLAS-AI = 0.90(VLAS-M)+0.20, Pearson’s linear correlation coefficient r = 0.873, n = 981. Blue line: fitted regression line, shading: 95% confidence interval, red dashed line: identity. Bland–Altman analysis (B) demonstrated a mean bias of 0.01 vertebrae (95% CI −0.003 to 0.03) for the VHS-AI method. Dashed lines give limits of agreement (±1.96 SD) and mean bias; shading gives 95% confidence intervals with dotted lines at the limits. VLAS indicates vertebral left atrial size; VLAS-AI indicates VLAS measured by RadAnalyzer, VLAS-M, VLAS measured by a human observer and SD, standard deviation.

**Fig 5:**
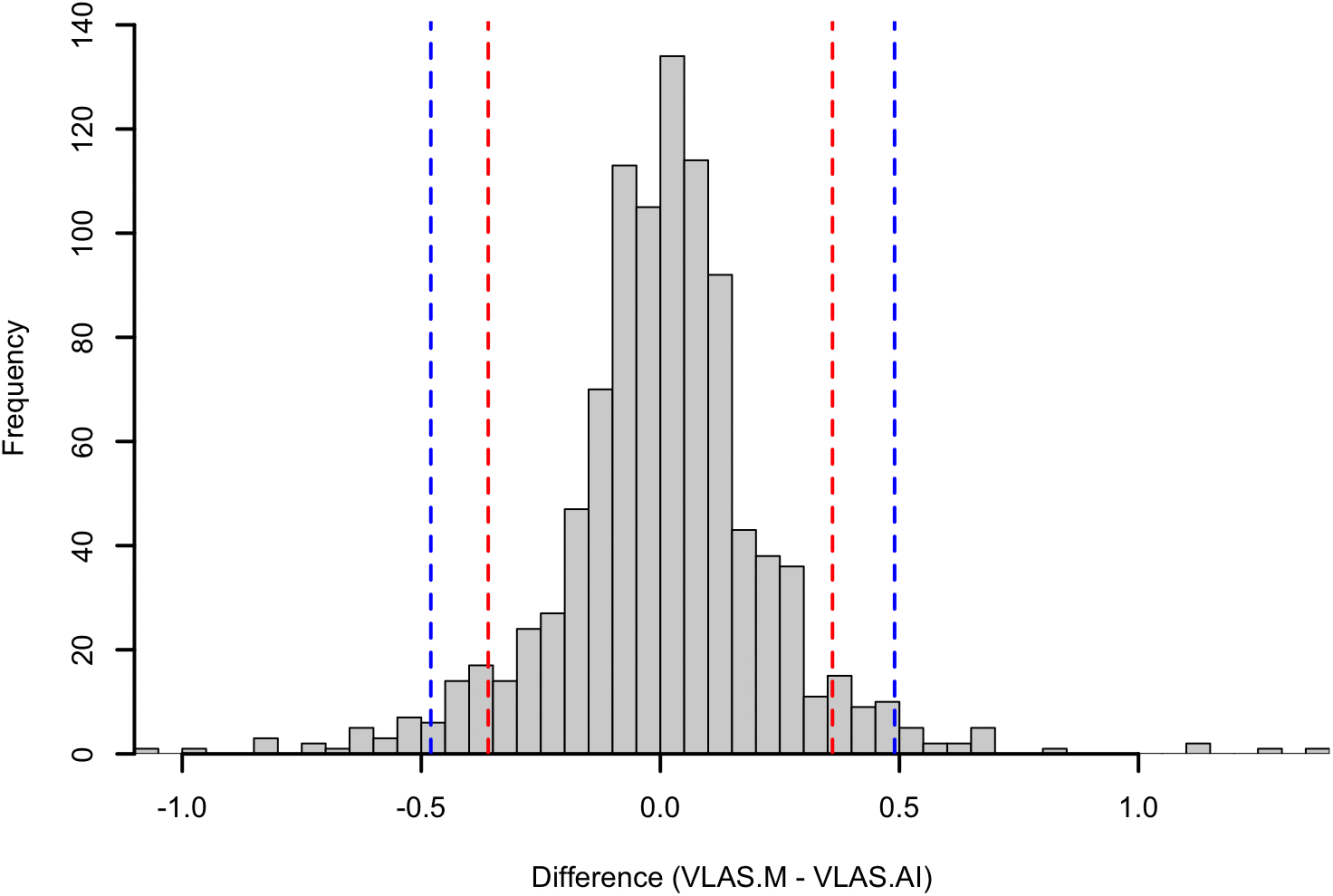
Histogram of the difference between modified vertebral left atrial size (VLAS) determined by human observer (VLAS.M) and Radanalyzer (VLAS.AI). The red lines indicate the 5th percentile and 95th percentile. The blue dashed lines represent the 2.5th percentile and the 97.5th percentile.

981 measurements of VLAS-M and VLAS-AI were available from right lateral radiographic views and Pearson’s correlation was 0.873 (95% CI 0.857 to 0.887 p < 0.001). Passing-Bablok analysis (Fig 4A) yielded the equation VLAS-AI = 0.90(VLAS-M)+0.20, with 95% CI of 0.88–0.93 for the slope and 0.13 to 0.25 for the intercept. Bland–Altman analysis (Fig 4B) showed a bias of 0.007 (SE 0.007; 95% CI −0.004 to 0.025) between VLAS-AI and VLAS-M with limits of agreement between −0.44 and 0.46 vertebrae although significant heteroscedasticity and proportional biases was observed. The 95^th^ percentile absolute difference between VLAS-M and VLAS-AI was 0.49 vertebrae (95% CI 0.44–0.51, Fig 5).

## Discussion

Deep-learning enabled software, RadAnalyzer, correlated better with trained observer for measuring VHS (r = 0.917, 95% CI 0.907 to 0.926) than VLAS (r = 0.873 (95% CI 0.857 to 0.887). The mean bias derived from Bland-Altman plots between AI and human for VHS and VLAS in this study were clinically insignificant, 0.002 and 0.007 vertebrae respectively. Based on the limits of agreement, 95% of the time results from humans and AI methods will be 0.85 vertebrae lower or 0.86 vertebrae higher for VHS and 0.44 vertebrae lower or 0.46 vertebrae higher for VLAS. These results were consistent for sub-analysis of Cavalier King Charles Spaniels and all other breeds. The mean bias for VHS is lower than a previous study using a different AI-algorithm to measure VHS where measurements differed by 0.09 vertebrae to [18]. Both AI algorithms give less variation than the 1.05 vertebrae that was observed between 16 human observers in another study [6].

Significant heteroscedasticity and proportional bias were observed for both VHS and VLAS, with RadAnalyzer tending to underestimate measurements in dogs with severe cardiomegaly. However, because this study included dogs across a wide range of disease severity—including the most severe cases—this degree of underestimation is unlikely to affect clinical decision-making. Thresholds for clinically relevant cardiomegaly (approximately 12.5 for VHS and 3.0 for VLAS) fall well within the observed measurement ranges (VHS 8.7–15.5; VLAS 1.0–5.0), making clinically meaningful misclassification unlikely [2–4].

RadAnalyzer can be deployed outside of a picture archiving and communication system (PACS) system and is accessible both on computers and via a smartphone application that captures images from any display. VHS and VLAS are time-consuming measurements and subject to interobserver variability, with performance generally improving with experience [6,9,22]. The goal of AI in veterinary medicine should be to support veterinarians, not replace them. By improving efficiency and measurement precision, AI tools such as RadAnalyzer may allow clinicians to devote more time to higher-value tasks, including diagnosis and patient management.

One of the key challenges in AI implementation is explainability. Although deep learning systems remain complex and are often considered “black boxes,” RadAnalyzer provides visual feedback by displaying landmarks used for VHS and VLAS measurements directly on the image. This allows clinicians to verify landmark placement and, if necessary, edit annotations with immediate recalculation of measurements. Additionally, RadAnalyzer does not generate measurements from non-diagnostic radiographs, reducing risk of overinterpretation of poor-quality images and supporting more reliable clinical decision-making.

If echocardiography is unavailable, radiographic indices of heart size, including VHS and VLAS, can be utilized for the diagnosis, staging and monitoring of canine cardiac disease [2,3,23]. This study was conducted because echocardiography is not readily available to many practicing veterinarians and there is variability between individuals when measuring VHS and VLAS [6,22]. Deep-learning technology eliminates inter-observer and intra-observer variability associated with human observers [6,22,24]. Reliable measurements and monitoring cardiac size is important as the rate of increase in VHS accelerates just before the onset of CHF in myxomatous mitral valve disease and a rate of change above >0.08 vertebral units per month is associated with the onset of CHF [25,26]. Similarly, a rate of change of increase in VLAS above 0.02 per month is associated with the onset of CHF in myxomatous mitral valve disease [27]. RadAnalyzer may therefore be particularly useful for repeated measurements and longitudinal monitoring.

There are several limitations to this study. A single observer obtained the manual VHS and VLAS measurements (VHS-M and VLAS-M), to reduce the impact of interobserver variability [6], intraobserver variability was not evaluated. This observer was also considered the ground truth, despite it being accepted that humans are inherently variable [6,12]. Future studies could compare agreement between radiographic measurements from RadAnalyzer with echocardiographic measurements of cardiac size as echocardiography is considered the most highly sensitive and specific method for the diagnosis and staging of canine cardiac disease and for measurements of cardiac size. This study also did not include patients with active heart failure, or other pulmonary infiltrate patterns as pulmonary opacities may affect performance. The effect of radiographic pulmonary infiltrates on the performance of the RadAnalyzer algorithm and use of RadAnalyzer in different clinical settings and populations could be investigated in future studies. Radanalyzer cannot distinguish between right and left lateral radiographs and has not been validated on left lateral radiographs.

## Conclusion

The commercially available web based and smartphone application, RadAnalyzer, generates comparable measurements of VHS and VLAS to a trained veterinarian on high quality radiographs without pulmonary infiltrates and will reduce the impact of interobserver variability. Following AI annotation and measurement, it is possible to view landmarks, enabling clinicians to confirm the validity or adjust the annotated landmarks associated with the measurement to avoid mistakes in clinical decision making, although this feature was not used in this study. Further studies are needed to compare agreement between measurements from this tool with echocardiographic variables, which may further support the use of this tool in making clinical decisions for patients with cardiac disease.

## Supporting information

S1 Table Dog breeds. Number and percentage of dogs of each breed included within the study.
(CSV)

S2Code R Code. (R)

S1 File Data. (CSV)

## References

[1] Keene BW, Atkins CE, Bonagura JD, Fox PR, Häggström J, Fuentes VL, et al. ACVIM consensus guidelines for the diagnosis and treatment of myxomatous mitral valve disease in dogs. J Vet Intern Med. 2019;33(3):1127–40. doi: 10.1111/jvim.15488 30974015 PMC6524084

[2] Poad MH, Manzi TJ, Oyama MA, Gelzer AR. Utility of radiographic measurements to predict echocardiographic left heart enlargement in dogs with preclinical myxomatous mitral valve disease. J Vet Intern Med. 2020;34(5):1728–33. doi: 10.1111/jvim.15854 32686167 PMC7517506

[3] Stepien RL, Rak MB, Blume LM. Use of radiographic measurements to diagnose stage B2 preclinical myxomatous mitral valve disease in dogs. J Am Vet Med Assoc. 2020;256(10):1129–36. doi: 10.2460/javma.256.10.1129 32364449

[4] Malcolm EL, Visser LC, Phillips KL, Johnson LR. Diagnostic value of vertebral left atrial size as determined from thoracic radiographs for assessment of left atrial size in dogs with myxomatous mitral valve disease. J Am Vet Med Assoc. 2018;253(8):1038–45. doi: 10.2460/javma.253.8.1038 30272515

[5] Buchanan JW, Bücheler J. Vertebral scale system to measure canine heart size in radiographs. J Am Vet Med Assoc. 1995;206(2):194–9. doi: 10.2460/javma.1995.206.02.194 7751220

[6] Hansson K, Häggström J, Kvart C, Lord P. Interobserver variability of vertebral heart size measurements in dogs with normal and enlarged hearts. Vet Radiol Ultrasound. 2005;46(2):122–30. doi: 10.1111/j.1740-8261.2005.00024.x 15869155

[7] Lamb CR, Tyler M, Boswood A, Skelly BJ, Cain M. Assessment of the value of the vertebral heart scale in the radiographic diagnosis of cardiac disease in dogs. Vet Rec. 2000;146(24):687–90. doi: 10.1136/vr.146.24.687 10887980

[8] Levicar C, Granados-Soler JL, Freise F, Raue JF, Nolte I, Bach J-P. Comparison of different radiographic scores with associated echocardiographic measurements and prediction of heart enlargement in dogs with and without myxomatous mitral valve disease. J Vet Cardiol. 2022;44:1–12. doi: 10.1016/j.jvc.2022.08.004 36174296

[9] Bagardi M, Manfredi M, Zani DD, Brambilla PG, Locatelli C. Interobserver variability of radiographic methods for the evaluation of left atrial size in dogs. Vet Radiol Ultrasound. 2021;62(2):161–74. doi: 10.1111/vru.12930 33226167

[10] Akinsulie OC, Idris I, Aliyu VA, Shahzad S, Banwo OG, Ogunleye SC, et al. The potential application of artificial intelligence in veterinary clinical practice and biomedical research. Front Vet Sci. 2024;11:1347550. doi: 10.3389/fvets.2024.1347550 38356661 PMC10864457

[11] Wilshaw J, Rosenthal SL, Wess G, Dickson D, Bevilacqua L, Dutton E, et al. Accuracy of history, physical examination, cardiac biomarkers, and biochemical variables in identifying dogs with stage B2 degenerative mitral valve disease. J Vet Intern Med. 2021;35(2):755–70. doi: 10.1111/jvim.16083 33645846 PMC7995403

[12] Burti S, Longhin Osti V, Zotti A, Banzato T. Use of deep learning to detect cardiomegaly on thoracic radiographs in dogs. Vet J. 2020;262:105505. doi: 10.1016/j.tvjl.2020.105505 32792095

[13] Li S, Wang Z, Visser LC, Wisner ER, Cheng H. Pilot study: Application of artificial intelligence for detecting left atrial enlargement on canine thoracic radiographs. Vet Radiol Ultrasound. 2020;61(6):611–8. doi: 10.1111/vru.12901 32783354 PMC7689842

[14] Solomon J, Bender S, Durgempudi P, Robar C, Cocchiaro M, Turner S, et al. Diagnostic validation of vertebral heart score machine learning algorithm for canine lateral chest radiographs. J Small Anim Pract. 2023;64(12):769–75. doi: 10.1111/jsap.13666 37622992

[15] Boissady E, De La Comble A, Zhu X, Abbott J, Adrien-Maxence H. Comparison of a deep learning algorithm vs. humans for vertebral heart scale measurements in cats and dogs shows a high degree of agreement among readers. Front Vet Sci. 2021;8:764570. doi: 10.3389/fvets.2021.764570 34957280 PMC8695672

[16] Malcolm EL, Gordon S, Haggstrom J. Esvc-o-33 variability of radiographic cardiac measurements measured by artificial intelligence-assisted software in dogs. In: 2025.

[17] Sánchez X, Prandi D, Badiella L, Vázquez A, Llabrés-Díaz F, Bussadori C, et al. A new method of computing the vertebral heart scale by means of direct standardisation. J Small Anim Pract. 2012;53(11):641–5. doi: 10.1111/j.1748-5827.2012.01288.x 23025353

[18] Sykes KT, Gordon S, Craig J, Vitt J, Rishniw M. Accuracy of deep learning-enabled software to measure vertebral heart size in dogs with myxomatous mitral valve disease. In: Journal of Veterinary Internal Medicine, 2020.

[19] Bronstein MM a B, LeCun Y, Szlam A, Vandergheynst P. Geometric Deep Learning: Going beyond Euclidean data. IEEE Signal Processing Magazine. 2017;34. doi: 10.1109/msp.2017.2693418

[20] Dietterich TG, editor Ensemble Methods in Machine Learning. 2000; Berlin, Heidelberg: Springer Berlin Heidelberg.

[21] Bland JM, Altman DG. Measuring agreement in method comparison studies. Stat Methods Med Res. 1999;8(2):135–60. doi: 10.1177/096228029900800204 10501650

[22] Levicar C, Nolte I, Granados-Soler JL, Freise F, Raue JF, Bach JP. Methods of radiographic measurements of heart and left atrial size in dogs with and without myxomatous mitral valve disease: intra- and interobserver agreement and practicability of different methods. Animals. 2022;12(19). doi: 10.3390/ani12192531 36230272 PMC9559670

[23] Lord P, Hansson K, Kvart C, Häggström J. Rate of change of heart size before congestive heart failure in dogs with mitral regurgitation. J Small Anim Pract. 2010;51(4):210–8. doi: 10.1111/j.1748-5827.2010.00910.x 20406369

[24] Vezzosi T, Puccinelli C, Tognetti R, Pelligra T, Citi S. Radiographic vertebral left atrial size: A reference interval study in healthy adult dogs. Vet Radiol Ultrasound. 2020;61(5):507–11. doi: 10.1111/vru.12896 32621373

[25] Lord PF, Hansson K, Carnabuci C, Kvart C, Häggström J. Radiographic heart size and its rate of increase as tests for onset of congestive heart failure in Cavalier King Charles Spaniels with mitral valve regurgitation. J Vet Intern Med. 2011;25(6):1312–9. doi: 10.1111/j.1939-1676.2011.00792.x 22092622

[26] Boswood A, Gordon SG, Häggström J, Vanselow M, Wess G, Stepien RL, et al. Temporal changes in clinical and radiographic variables in dogs with preclinical myxomatous mitral valve disease: The EPIC study. J Vet Intern Med. 2020;34(3):1108–18. doi: 10.1111/jvim.15753 32200574 PMC7255670

[27] Lee D, Yun T, Koo Y, Chae Y, Ku D, Chang D, et al. Change of Vertebral Left Atrial Size in Dogs With Preclinical Myxomatous Mitral Valve Disease Prior to the Onset of Congestive Heart Failure. J Vet Cardiol. 2022;42:23–33. doi: 10.1016/j.jvc.2022.05.003 35675727

